# MicroRNA Regulation of Human Herpesvirus Latency

**DOI:** 10.3390/v14061215

**Published:** 2022-06-02

**Authors:** Siyu Chen, Yue Deng, Dongli Pan

**Affiliations:** 1 State Key Laboratory for Diagnosis and Treatment of Infectious Diseases, The First Affiliated Hospital, Zhejiang University School of Medicine, Hangzhou 310030, China; 13467699406@163.com (S.C.); 21918017@zju.edu.cn (Y.D.); 2 Department of Medical Microbiology and Parasitology, Zhejiang University School of Medicine, Hangzhou 310030, China

**Keywords:** microRNA, herpesvirus, latency, reactivation, HSV, HCMV, EBV, KSHV

## Abstract

Herpesviruses are ubiquitous human pathogens. After productive (lytic) infection, all human herpesviruses are able to establish life-long latent infection and reactivate from it. Latent infection entails suppression of viral replication, maintenance of the viral genome in infected cells, and the ability to reactivate. Most human herpesviruses encode microRNAs (miRNAs) that regulate these processes during latency. Meanwhile, cellular miRNAs are hijacked by herpesviruses to participate in these processes. The viral or cellular miRNAs either directly target viral transcripts or indirectly affect viral infection through host pathways. These findings shed light on the molecular determinants that control the lytic-latent switch and may lead to novel therapeutics targeting latent infection. We discuss the multiple mechanisms by which miRNAs regulate herpesvirus latency, focusing on the patterns in these mechanisms.

## 1. Introduction

Herpesviruses are ubiquitous double-stranded DNA viruses infecting a wide range of animal hosts. In humans, there are nine known herpesviruses within the family of Herpesviridae, including herpes simplex virus 1 (HSV-1), HSV-2 and varicella zoster virus (VZV) in the alphaherpesvirus subfamily, human cytomegalovirus (HCMV), human herpesvirus 6A (HHV-6A), HHV-6B and HHV-7 in the betaherpesvirus subfamily, and Epstein–Barr virus (EBV) and Kaposi’s sarcoma-associated herpesvirus (KSHV) in the gammaherpesvirus subfamily. Although these viruses mostly cause asymptomatic infections or self-limited diseases, they can occasionally lead to fatal outcomes especially for immunocompromised patients and newborns. Moreover, none of the herpesvirus-associated diseases is currently curable because of the ability of these viruses to establish life-long latent infection and then to reactivate from it. The latency-reactivation cycle allows the virus to indefinitely reside inside the host body with the potential to propagate whenever the situation is advantageous to the virus. It is key to the success of herpesviruses in achieving high prevalence in the human population.

Different herpesviruses prefer different cell types for latency [1]. Alphaherpesviruses establish latency in neurons. Among betaherpesviruses, HCMV establishes latency primarily in CD34+ hematopoietic progenitor cells (HPCs), and HHV-6 and HHV-7 establish latency mainly in T lymphocytes. Gammaherpesviruses establish latency in B lymphocytes. Despite these differences, different herpesviruses follow shared principles to undergo latency. First, viral genes associated with the productive (lytic) cycle are silenced so that there is basically no production of infectious virus during latency. Second, the viral genome persists intact in the host cell. Third, when stimulated, the viral genome can reactivate to resume production of infectious virus. Therefore, all forms of latency entails maintenance of the viral genome with alteration of lytic and latent programs of viral gene expression.

Human herpesvirus genomes are typically maintained as episomes in the nucleus except that HHV-6 genomes can sometimes integrate into the host chromosome [2]. Such genomes are usually stable. However, suppression of viral antigen expression is not absolute, as the viruses need to be poised for reactivation. Therefore, latently infected cells are not entirely invisible to the host immune system, so the viruses need to possess strategies that prevent the cells from being recognized or cleared by the immune system even during latency.

The lytic and latent programs of herpesvirus infection have quite different gene expression profiles. During lytic infection, viral gene expression proceeds in a cascade fashion with immediate-early (IE), early, and late genes being sequentially expressed, whereas during latent infection, viral gene expression is usually limited to a set of noncoding RNAs and at most a few antigens. Chromatin-mediated transcriptional regulation contributes substantially to the remarkable difference in the viral gene expression profiles between lytic and latent infection [3]. Herpesvirus genes are generally associated with activating chromatin modifications during lytic infection. However, during latency, herpesvirus genomes mostly exist in a nucleosomal state associated with repressive histone modifications, with only small regions associated with activating histone modifications, while chromatin insulators often serve as boundaries to separate active and inactive regions of the genomes.

Besides transcriptional regulation, post-transcriptional regulation of gene expression by microRNAs (miRNAs) have been demonstrated to play an important role in regulating the lytic–latent balance. miRNAs are derived from polyadenylated primary transcripts that are cleaved by Drosha in the nucleus, giving rise to hairpin-shaped precursor miRNAs (pre-miRNAs). Pre-miRNAs are exported to the cytoplasm and further cleaved by Dicer to produce the ~22 nt double-stranded RNAs. One strand of the duplex known as the mature strand is incorporated into the RNA-induced silencing complex (RISC), where binding of the miRNA to the target mRNA mediates mRNA cleavage or decay or translation inhibition. While miRNAs can bind to all regions of mRNAs, efficacious gene regulation usually results from binding to the 3′ untranslated regions (UTRs). The miRNA seed region (nucleotides 2–8) is critical for target recognition although sequences outside the seed region sometimes contribute [4]. Because of the short sequences required for miRNA targeting, each miRNA can target multiple genes, and a single gene can be targeted by multiple miRNAs, resulting in complex gene regulation networks. It is predicted that most mammalian mRNAs are conserved targets of miRNAs [5]. Although the impact of a single miRNA-mRNA interaction is often limited, the collective impact of regulating multiple targets of a single miRNA can be profound, as demonstrated by a variety of major defects exhibited by miRNA knockout mice [4].

In addition to their role in normal cellular functions, miRNAs also participate in regulation of viral infection [6,7]. miRNAs are ideal tools for viruses to manipulate the cellular environment in favor of viruses because they are non-immunogenic yet have the capacity to broadly perturb gene expression landscapes. Moreover, their short sequences would not take much genomic space, so encoding them in viruses would cause little burden to the viral packaging capacity. Herpesviruses are particularly good at utilizing miRNAs. Currently, at least 299 herpesvirus miRNAs have been identified [8]. Latency is a major process regulated by these miRNAs [9]. Since miRNAs usually require hours to days to significantly alter protein levels, the impact of miRNAs on rapid processes is presumably limited, but given enough time, the accumulative effects on lifelong latent or persistent infection can be profound. Interestingly, many herpesvirus miRNAs are among the only few gene products highly expressed during latency. Besides viral miRNAs, the role of host miRNAs in herpesvirus latency is being increasingly recognized. Viral and host miRNAs are involved in various processes during latency, including silencing of lytic genes, cell survival and proliferation, immune evasion, and reactivation.

## 2. Herpesvirus miRNA Expression during Latency

**Figure 1:**
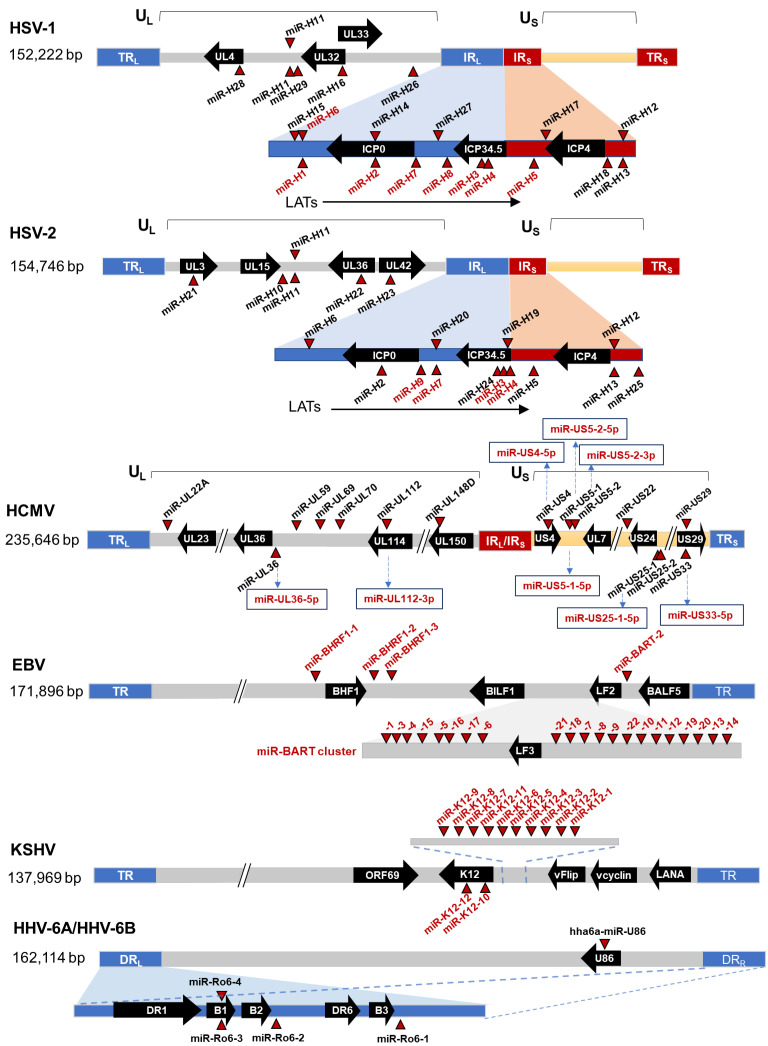
Genomic locations of human herpesvirus miRNAs. The locations of miRNA precursors are denoted by red triangles. Red characters indicate the miRNAs expressed during latent infection. Thick arrows along the genomes represent selected viral genes and the directions of transcription.

Genomic locations of human herpesvirus miRNAs are illustrated in Figure 1. Currently, 20 HSV-1 and 18 HSV-2 miRNAs have been identified [10,11,12,13,14,15], among which nine are conserved in position and/or sequence between HSV-1 and HSV-2 [10]. Many of these miRNAs, including miR-H2, H3, H4, H5, and H7 of both HSV-1 and HSV-2; miR-H8 of HSV-1; and miR-H9, H10, and H24 of HSV-2 are clustered within the latency-associated transcript (LAT) region, while miR-H1 and H6 are located nearby. Expression of miR-H2, H3, H4, H5, and H6 is high and dependent on the LAT promoter in HSV-1 latently infected mouse trigeminal ganglia (TG) [16]. Like LAT, their levels decrease during explant-stimulated reactivation [17]. HSV-1 miR-H2 through miR-H8 are also detectable in latently infected human TG [18,19,20]. Therefore, expression of these miRNAs is likely driven by the LAT promoter [21]. HSV-1 lytic infection inhibits pre-miRNA processing, but the inhibition is alleviated during latency, which, combined with high LAT promoter activity, permits high expression of these miRNAs consistent with their role in HSV latency [22]. The situation is quite different for the other alphaherpesvirus, VZV. Analysis of human TG obtained from autopsy that readily detected VZV DNA failed to identify any VZV miRNAs [18]. One study identified 24 potential VZV miRNAs in lytically infected fibroblasts and human embryonic stem cell-derived (hESC) neurons, but almost no sequence was detected in latently infected hESC neurons although latent expression of one of the putative miRNAs was shown by qRT-PCR [18].

For betaherpesviruses, HCMV encode at least 22 mature miRNAs derived from 12 pre-miRNAs [23,24,25,26]. Unlike alpha and gamma herpesviruses, HCMV miRNAs are scattered throughout the genome. Examination of their expression in latently infected monocytes, THP-1 cells, or CD34+ HPCs showed expression of many of these miRNAs during latent infection, including miR-UL112, miR-UL36, miR-UL22A, miR-US5-1, miR-US5-2, miR-US25-1, miR-US25-2, etc. [27,28,29]. Eight HHV-6B mature miRNAs derived from four pre-miRNAs have been identified and are conserved in HHV-6A [30]. Another study identified an additional HHV-6A miRNA not conserved with HHV-6B [31]. Whether these miRNAs are expressed during latency is unknown. There has been no report on miRNA expression by HHV-7.

Regarding gammaherpesviruses, EBV encode at least 44 mature miRNAs derived from 25 pre-miRNAs. Four of these mature miRNAs are located within the BHRF1 region and designated BHRF1 miRNAs [32,33]. The other 40 miRNAs are located in the BART region and designated BART miRNAs [34,35]. Based on viral gene expression patterns, EBV latency is classified into type 0, 1, 2, and 3 latency [1]. EBV miRNAs are differentially expressed during different latency types [36]. BHRF1 miRNAs are expressed mainly during type 3 latency, whereas BART miRNAs are expressed in all types of latency [37]. KSHV encodes at least 13 pre-miRNAs, which are processed to yield 25 mature miRNAs. Most of these miRNAs are located in the latent-associated region and expressed during latency [38,39].

## 3. Viral miRNAs Directly Targeting Viral Lytic Transcripts

**Figure 2:**
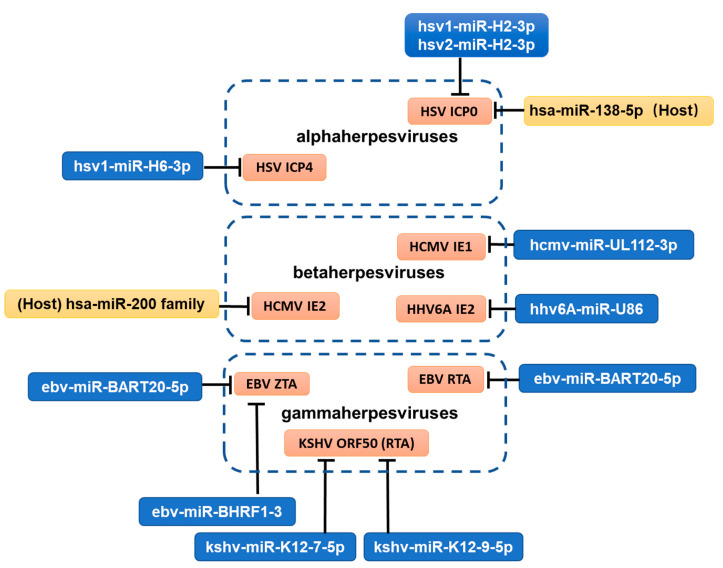
Direct targeting of viral IE genes by viral and cellular miRNAs. Blue rectangles represent viral miRNAs, and yellow rectangles represent host ones.

To establish latency, viral lytic genes need to be silenced. Multiple important lytic genes, predominantly IE genes, are direct targets of viral miRNAs (Figure 2, Table 1). Some miRNAs even exhibit perfect base pairing with their targets due to their genomic locations being antisense to their targets. This pattern might represent a strategy of the viruses to impede the onset of the lytic cycle during establishment of latency.

HSV-1 and HSV-2 miR-H2-3p are antisense to the coding region of the ICP0 transcript and repress ICP0 expression in co-transfection assays [10,14,40]. Likewise, miR-H3-3p and H4-5p of both HSV-1 and HSV-2 are perfectly complementary to the ICP34.5 transcript and repress ICP34.5 expression in co-transfection assays [11,13,41]. Binding of HSV-1 miR-H2-3p to ICP0 mRNA and binding of miR-H4-5p (but not miR-H3) to ICP34.5 mRNA have been confirmed by photoactivatable ribonucleoside-enhanced crosslinking and immunoprecipitation (PAR-CLIP) [41]. ICP0 is an IE protein crucial for initiation of viral lytic gene expression, and ICP34.5 is a neurovirulence factor. Reducing lytic gene expression and neurovirulence should favor latency. However, these miRNAs are not essential for latency in animal models. Although one study showed increased reactivation exhibited by a miR-H2 HSV-1 mutant [42], this phenotype was not observed with another miR-H2 mutant [40]. Furthermore, HSV-2 mutants with mutations in miR-H2, H3, or H4 showed little difference from the rescued viruses in viral DNA and RNA levels during latency and in reactivation kinetics in a guinea pig model [43]. These results may reflect functional redundancy of viral miRNAs. For both HSV-1 and HSV-2, miR-H6-3p is predicted to target the essential viral transcription factor ICP4. HSV-1 miR-H6-3p was shown to repress ICP4 expression in co-transfection assays [11], but HSV-2 miR-H6-3p had no detectable effect on ICP4 expression during transfection or infection [44].

HCMV miR-UL112-3p targets IE1, a crucial viral transactivator of lytic gene expression and inhibits HCMV replication [45,46]. Deleting the miR-UL112-3p target site resulted in an increase in the IE1 RNA level in HCMV latently infected primary monocytes [47]. Similarly, HHV-6A miR-U86, encoded in the opposite strand of the U86 gene that encodes the viral IE2 gene, represses viral replication by targeting the IE2 transcript [31].

EBV ZTA (BZLF1) and RTA (BRLF1) are IE proteins with transactivating activities critical for lytic replication. miR-BART20-5p targets both BZLF1 and BRLF1 transcripts through their shared 3′ UTR [48]. miR-BHRF1-3 also targets the BZLF1 3′ UTR and attenuates lytic replication following reactivation [49]. The homolog of EBV RTA in KSHV is ORF50, which is directly targeted by KSHV miR-K12-7-5p and miR-K12-9-5p and indirectly suppressed by miR-K12-5 [50,51]. Notably, a specific antagonism of miR-K12-9-5p in latently infected cells enhances the frequency of spontaneous reactivation [50]. Besides RTA and ZTA, EBV DNA polymerase BALF5 is also a target of its own miRNA miR-BART2-5p [32,52].

## 4. Viral miRNAs Repressing Cellular Pathways Important for Viral Replication

Several herpesvirus miRNAs do not directly target viral transcripts but can still restrict lytic infection by targeting cellular pathways that can activate viral replication thereby potentially promoting latency. Interestingly many of the cellular pathways eventually act on IE gene expression, highlighting the importance of repressing initiation of lytic gene expression for establishment of latency.

HCMV miR-U148D targets IER5, which was identified as a positive regulator of HCMV lytic replication [53]. IER5 transcriptionally represses CDC25B, which in turn suppresses HCMV IE1 and lytic gene transcription by activating cyclin-dependent kinase 1 (CDK1). Therefore miR-U148D might favor HCMV latency by modulating the IER5-CDC25B-CDK1 signaling pathway.

Dicer was identified as a positive regulator of the EBV lytic cycle [54]. Its repression by miR-BART6 leads to repression of important IE genes such as RTA and ZTA. EBV miR-BART-18-5p targets MAP kinase 2 (MAP3K2), which is an intermediary in the signaling pathways that initiate EBV lytic viral replication [55]. EBV miR-BHRF1-2-5p and miR-BART2-5p target genes related to B-cell receptor (BCR) signaling [56]. Since BCR engagement can trigger EBV replication, these miRNAs restrict reactivation from latency, as has been demonstrated by the enhancement of BCR-mediated reactivation following inhibition of these miRNAs.

KSHV miR-K12-1-5p targets IκBα, an inhibitor of NF-κB complexes, to rescue NF-κB activity and inhibit viral replication [57]. miR-K12-3-5p can suppress KSHV lytic replication and gene expression by targeting nuclear factor I/B, which is a cellular activator of the viral ORF50 (RTA) promoter [58].

## 5. Cellular miRNAs Directly Targeting Viral Lytic Transcripts

Given that viruses often hijack host molecules for various viral purposes, it is not surprising that host miRNAs are exploited by herpesviruses to facilitate latency. Having their important IE genes be targeted by cellular miRNAs abundant in latently infected cells is a straightforward way of achieving this (Figure 2). One such example comes from our studies with HSV, which undergoes lytic infection in a wide variety of cell types but specifically establishes latency in neurons. We found that neuron-specific miR-138-5p targets both HSV-1 and HSV-2 ICP0 [59]. The target sites are partially conserved among HSV-1, HSV-2, and chimpanzee herpesvirus 1. Repression of ICP0 by miR-138-5p is functionally conserved but mechanistically different between HSV-1 and HSV-2 [60]. Binding of miR-138-5p to both HSV-1 and HSV-2 ICP0 transcripts was confirmed by PAR-CLIP. For both HSV-1 and HSV-2, mutant viruses with disrupted binding to miR-138-5p showed increased ICP0 expression in neuronal cells. Moreover, in a mouse model, the HSV-1 mutant showed not only increased lytic gene expression in TG during both establishment and maintenance of latency but also increased host mortality [59]. Notably since miR-H2-3p also targets ICP0, HSV appears to employ both viral and cellular miRNAs to ensure that this important lytic gene is silenced during latency.

An analogous situation was observed with HCMV. The HCMV UL122 transcript that encodes the important IE2 protein is targeted by the miR-200 family [61]. Mutation of the miR-200 target sites in UL122 resulted in increased lytic gene expression and virus replication in an in vitro HCMV latency model. Interestingly, miR-200 levels are high in cells that favor HCMV latency, but the levels decrease as the cells differentiate into those permissive for lytic infection, indicating that the change in miR-200 expression might contribute to the lytic-latent switch.

## 6. Cellular miRNAs Repressing Cellular Pathways Important for Viral Replication

Cellular miRNAs might also target cellular genes important for viral replication. Such miRNA-target interactions exist in uninfected cells and should have their own cellular functions. However, during evolution, viruses might have evolved to take advantage of such interactions to facilitate latency. An example has to do with the aforementioned miR-138-5p, which not only targets HSV ICP0 but also targets host transcripts encoding transcription factors OCT-1 and FOXC1 [62]. Correlating with high neuronal expression of miR-138-5p, expression of OCT-1 and FOXC1 is low in neuronal cells. OCT-1 is an HSV VP16 co-factor that mediates activation of viral IE gene transcription by binding to IE gene promoters [63]. FOXC1 is a newly identified host activator of replication of both HSV-1 and HSV-2 in neuronal cells [60,62]. FOXC1 broadly promotes HSV lytic gene expression by reducing heterochromatin on viral genes at early times of infection. Therefore, it happens that miR-138′s viral and host targets, ICP0, OCT-1, and FOXC1, are all strong activators of HSV IE gene expression. Targeting these genes simultaneously should robustly repress lytic genes during latency.

Another example came from a study with EBV. As mentioned above, Dicer is a positive regulator of EBV lytic replication targeted by a viral miRNA. Interestingly, Dicer is also targeted by a host miRNA, let-7a [64]. During EBV latent infection let-7a expression is elevated by the EBV protein EBNA1. Probably due to the regulation of Dicer, a let-7a mimic decreased the percentage of EBV latently infected cells that reactivated to the lytic cycle, while a let-7a sponge had the opposite effect suggesting that let-7a helps maintain EBV latency.

## 7. Induction and Mimicry of Cellular miRNAs to Promote Cell Proliferation

**Figure 3:**
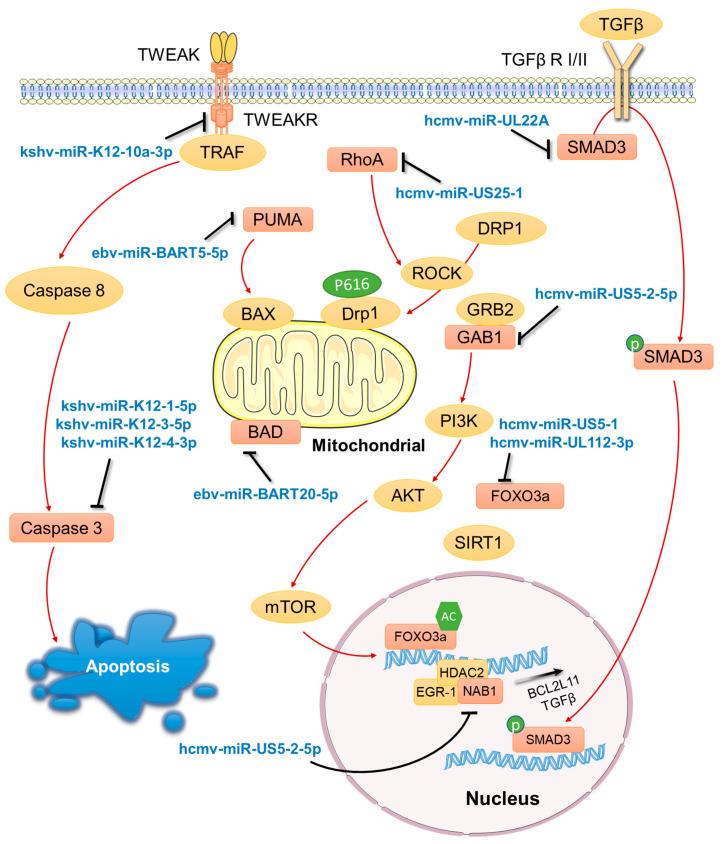
Regulation of apoptosis pathways by viral and cellular miRNAs. The genes directly targeted by miRNAs are indicated by orange rectangles. Green patterns represent protein modification. Some graphic elements were obtained from Servier Medical Art (SMART) (https://smart.servier.com/ (accessed on 12 May 2022)).

Besides silencing of lytic genes, viral latency also entails maintenance of cells harboring the latent genomes. While survival of infected neurons that do not divide may be sufficient for alphaherpesviruses, beta and gamma herpesviruses need their host cells to keep dividing since these cells have limited lifespans. Gammaherpesviruses are particularly good at using miRNAs to promote B-cell proliferation and these mechanisms may contribute to viral latency and oncogenesis (Figure 3). One strategy of EBV is induction of a cellular oncomiR, miR-155-5p [65]. In EBV latently infected B cells, miR-155-5p is strongly upregulated in part due to transcriptional activation through the NF-κB pathway by the viral LMP1 protein [66]. Upregulated miR-155-5p promotes lymphoblastoid cell growth and inhibiting apoptosis [67]. miR-155-5p also targets IKKε and contributes to EBV persistence by modulating NF-κB signaling and suppressing host innate immunity [68].

Intriguingly, some viral miRNAs mimic host miRNAs so that they can exploit the existing cellular target networks. KSHV miR-K12-11-3p is a miR-155-5p analog and shares 100% seed sequence homology with miR-155 [69]. miR-K12-11-3p and miR-155-5p co-target an extensive set of genes associated with pro-apoptotic and cell-cycle-regulatory functions [70]. Indeed, both miR-K12-11-3p and miR-155-5p induced B-cell proliferation in the spleen in an in vitro model where CD34+ human cord blood cells transduced with either miRNA were engrafted into humanized mice [71]. As another example, a systematic study based on high-throughput sequencing and crosslinking immunoprecipitation (HITS-CLIP) experiments in EBV-transformed B cells revealed 1774 human transcripts targeted by EBV miRNAs [72]. Half of them are also targets of the oncogenic miR-17-92 miRNA cluster, many of which are associated with apoptosis and the cell cycle, raising the possibility that EBV express miRNAs that functionally mimic this cluster for optimal maintenance of infected cells.

## 8. Other Viral miRNAs Regulating Apoptosis and Cell Proliferation

In addition to mimicking cellular miRNAs, there is additional evidence that gammaherpesviruses miRNAs block apoptosis and promote cell proliferation (Figure 3). One study using recombinant viruses lacking expression of EBV miR-BHRF miRNAs concluded that miR-BHRF miRNAs promote cell-cycle progression and prevent apoptosis of EBV-infected B cells [73,74]. Another study found that EBV miR-BART miRNAs sustain Burkitt’s lymphoma cells in the absence of other viral oncogenes as well as promoting the transformation of primary B lymphocytes [75]. Burkitt’s lymphoma cells engineered to lose EBV died by apoptosis and could be rescued by expressing viral miRNAs in them. Regarding KSHV, deletion of a cluster of ten KSHV miRNAs resulted in cell-cycle arrest and apoptosis of KSHV transformed primary rat mesenchymal precursor cells, which can be explained by redundant targeting of cell growth and survival pathways by some of these miRNAs [76]. Studies focusing on individual miRNAs also found that many of them target apoptosis pathways. For example, miR-BART5-5p targets p53 up-regulated modulator of apoptosis (PUMA) [77]. miR-BART20-5p targets the BCL2-associated agonist of cell death (BAD), a mediator of caspase-3-dependent apoptosis [78,79]. KSHV miR-K12-1-5p, miR-K12-3-5p, and miR-K12-4-3p target Caspase 3, a central mediator of apoptosis [80]. miR-K12-10a-3p targets tumor necrosis factor (TNF)-like weak inducer of apoptosis (TWEAK) receptor (TWEAKR) [81].

HCMV also use miRNAs to regulate apoptosis and cell proliferation during latency. HCMV miR-US5-1 and miR-UL112-3p target the proapoptotic transcription factor FOXO3a after infection of CD34+ HPCs, resulting in reduced expression of the proapoptotic BCL2L11 transcript [82]. As a different type of regulation, HCMV miR-US5-2-5p increases TGF-β production by targeting the transcriptional repressor NGFI-A binding protein (NAB1) to induce myelosuppression of uninfected CD34+ HPCs [83]. However high concentrations of TGF-β negatively affect CD34+ HPC growth and proliferation; therefore, to protect infected cells from the negative effects of TGF-β signaling, HCMV miR-UL22A blocks TGF-β signaling by downregulating SMAD3, and this mechanism is required for maintenance of HCMV latency. Cell proliferation may not always favor HCMV latency, as evidenced by one study that showed that HCMV miR-US25-1 targets RhoA, a small GTPase required for CD34+ HPC self-renewal and proliferation [84]. Infection with an HCMV mutant lacking miR-US25-1 resulted in increased proliferation of CD34+ HPCs but decreased the proportion of genome-containing cells in latency culture, indicating that miR-US25-1 contribute to viral genome maintenance by preventing excessive cell proliferation.

## 9. Viral miRNAs Antagonizing Innate and Adaptive Immunity

**Figure 4:**
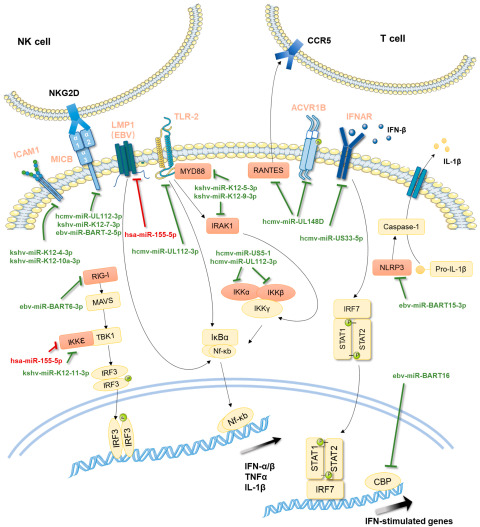
Regulation of host immune pathways by viral and cellular miRNAs. The genes directly targeted by miRNA are indicated by orange rectangles or characters. The red and green characters represent host and viral miRNAs, respectively. Some graphic elements were obtained from Servier Medical Art (SMART) (https://smart.servier.com/ (accessed on 12 May 2022)).

Latency itself is a viral immune evasion strategy. As further assurance, herpesviruses utilize miRNAs to interfere with various immune processes during latency (Figure 4). This strategy is particularly widely used by beta and gamma herpesviruses. One mechanism related to the regulation of NK-cell-mediated innate immune responses is shared by multiple herpesviruses: HCMV miR-UL112-3p, KSHV miR-K12-7-3p, and EBV miR-BART-2-5p all directly target the stress-induced immune ligand MICB to escape recognition by NK cells [85,86,87].

Other mechanisms are different for different viruses, but they generally involve regulation of the signaling pathways related to the production and functions of interferons or inflammatory cytokines. For HCMV, miR-UL148D targets the cellular receptor ACVR1B of the activin signaling axis in latently infected monocytes and represses expression of the downstream proinflammatory cytokine IL-6 [88]. miR-UL148D also targets the cytokine RANTES, which has a function of attracting immune cells during immune responses [89]. miR-UL112-3p, US5-1, and US5-2-5p target multiple components of the host secretory pathway important for the release of inflammatory cytokines, including VAMP3, RAB5C, RAB11A, SNAP23, and CDC42 [90]. miR-UL112-3p targets Toll-like receptor 2 and modulates the downstream TLR2/IRAK1/NFκB immune signaling pathway in THP-1 cells [91]. miR-US5-1 and miR-UL112-3p target the IκB kinase (IKK) complex components IKKα and IKKβ to limit production of proinflammatory cytokines [92]. miR-US33as-5p targets interferon alpha and beta receptor subunit 1 (IFNAR1) and confers IFN resistance during both lytic and latent infection [93].

For EBV, miR-BART16 targets cAMP response element-binding protein (CBP), a transcriptional coactivator in IFN signaling, and inhibits the production of IFN-stimulated genes in response to IFN-α stimulation [94]. miR-BART6-3p, which is highly expressed in latency II malignancy, targets an important pattern recognition receptor RIG-I to inhibit IFN-β production [95]. miR-BART15-3p targets NLRP3, a key component of the NLRP3 inflammasome complex [96].

Proteomics screening of human targets of KSHV miRNAs identified that miR-K12-4-3p and miR-K12-10a-3p indirectly decrease lysophosphatidic acid-stimulated ICAM1 expression, which could potentially minimize recruitment of leukocytes to areas of KSHV infection [97]. miR-K12-9-3p and miR-K12-5-3p target IRAK1 and MYD88, respectively, both of which are components of the Toll-like receptor/Interleukin-1R signaling cascade [98]. miR-K12-11-3p can attenuate IFN signaling by targeting IKKε during latent infection and inhibit KSHV reactivation induced by vesicular stomatitis virus [99].

Besides innate immunity, T-cell-mediated immunity is also modulated by herpesvirus miRNAs, as has been shown for EBV. By comparing an EBV mutant expressing a set of miRNAs with a mutant expressing no miRNA, a study showed that EBV miRNAs interfere with various processes leading to activation of EBV-specific CD4+ effector T cells, including release of proinflammatory cytokines, differentiation of naive CD4+ T cells to Th1 cells, and peptide processing and presentation [100]. Another study conducted in a similar manner showed that EBV miRNAs also counteract immune surveillance by CD8+ T cells by direct targeting of the peptide transporter subunit TAP2 and reducing levels of the TAP1 subunit and MHC class I molecules [101]. Consistently, in a humanized mouse model, mice infected with miRNA-deficient EBV replicated to lower viral titers with decreased frequencies of proliferating EBV-infected B cells relative to WT EBV, whereas when CD8+ T cells were depleted, the miRNA-deficient virus reached similar viral loads as wild-type EBV, suggesting that EBV miRNAs play an important role in immune evasion from CD8+ T cells [102].

## 10. Viral and Cellular miRNAs That Promote Reactivation

There have been only a few examples of miRNAs promoting reactivation from latency. These miRNAs are usually highly expressed during lytic infection or reactivation. HSV-1 miR-H1 and miR-H6 are located in opposite strands of a common sequence. Deletion of this sequence from HSV-1 resulted in impaired reactivation in mouse and rabbit models, implying that the two miRNAs together play a role in reactivation [103]. HCMV miR-US22-5p directly targets EGR1, and an HCMV miR-US22-5p mutant fails to reactivate in CD34+ HPCs [29]. Since EGR-1 promotes CD34+ HPC self-renewal, but reactivation usually requires differentiation, downregulation of EGR-1 may induce a cellular differentiation pathway necessary for HCMV reactivation. Additionally, by targeting the EGFR adaptor protein GAB1 and attenuating MEK/ERK signaling, HCMV miR-US5-2-5p indirectly represses the expression of EGR-1 as well as an important latency-associated viral gene UL138, thereby potentially playing a role in reactivation from latency [104].

Regarding cellular miRNAs, the miR-200 family members, namely miR-200b-3p and miR-429, target ZEB1 and ZEB2, which are suppressors of BZLF1 expression. These miRNAs promote reactivation in a ZEB-dependent manner [105]. Expression of these miRNAs is low in latently infected cells but elevated in plasma cells [106], indicating that induction of these miRNAs might represent a switch from latency to reactivation.

**Table 1:** miRNAs with potential role in herpesvirus latency.

Role in Latency	Viral or Cellular miRNAs	Mature miRNAs	Targets (Viral or Host)	References
Repression of viral lytic genes	Viral	hsv1-miR-H2-3p hsv2-miR-H2-3p	ICP0 (viral)	[10,14,40]
		hsv1-miR-H3-3p hsv1-miR-H4-5p	ICP34.5 (viral)	[11,13,41]
		hsv1-miR-H6-3p	ICP4 (viral)	[11]
		hcmv-miR-UL112-3p	HCMV IE1 (viral)	[45,46]
		hhv6a-miR-U86	HHV6A IE2 (viral)	[31].
		ebv-miR-BART20-5p	EBV ZTA (viral)	[48]
			EBV RTA (viral)	[48]
		ebv-miR-BART2-5p	EBV BALF5 (viral)	[32,52]
		kshv-miR-K12-9-5p	KSHV ORF50 (viral)	[50]
		kshv-miR-K12-7-5p	KSHV ORF50 (viral)	[50,51].
		ebv-miR-BHRF1-3	EBV ZTA (viral)	[49]
	Cellular	hsa-miR-138-5p	HSV ICP0 (viral)	[59]
		hsa-miR-200 family	HCMV UL122 (viral)	[61]
Repression of cellular pathways important for viral replication	Viral	hcmv-miR-U148D	IER5 (host)	[53]
		ebv-miR-BART6-5p	DICER (host)	[54]
		ebv-miR-BART-18-5p	MAP3K2 (host)	[55]
		ebv-miR-BHRF1-2-5p	BCR (host)	[56]
		ebv-miR-BART2-5p	BCR (host)	[56]
		kshv-miR-K12-1-5p	IκBα (host)	[57]
		miR-K12-3-5p	nuclear factor I/B (host)	[58]
	Cellular	hsa-miR-138-5p	FOXC1 (host)	[60]
			OCT-1 (host)	[60]
		hsa-let-7a	DICER (host)	[64]
Modulation of apoptosis and cell proliferation	Viral	kshv-miR-K12-11-3p	N.D.	[70,71]
		ebv-miR-BART5-5p	PUMA (host)	[77]
		ebv-miR-BART20-5p	BAD (host)	[78,79]
		kshv-miR-K12-1-5p	Caspase 3 (host)	[80]
		kshv-miR-K12-3-5p	Caspase 3 (host)	[80]
		kshv-miR-K12-4-3p	Caspase 3 (host)	[80]
		kshv-miR-K12-10a-3p	TWEAKR (host)	[81]
		hcmv-miR-US5-1	FOXO3a (host)	[82]
		hcmv-miR-UL112-3p	FOXO3a (host)	[82]
		hcmv-miR-US5-2-5p	NAB1 (host)	[83]
		hcmv-miR-UL22A	SMAD3 (host)	[83]
		hcmv-miR-US25-1	RhoA (host)	[84]
	Cellular	hsa-miR-155-5p	LMP1 (viral)	[66]
			IKKε (host)	[68]
		hsa-miR-17-92 cluster	N.D.	[72]
Antagonism of innate and adaptive immunity	Viral	hcmv-miR-UL112-3p	MICB (host)	[85,86]
		kshv-miR-K12-7-3p	MICB (host)	[87]
		ebv-miR-BART-2-5p	MICB (host)	[87]
		hcmv-miR-UL148D	ACVR1B (host)	[88]
			RANTES (host)	[89]
		hcmv-UL112-3p hcmv-US5-1 hcmv-US5-2-5p	VAMP3 (host)	[90]
			RAB5C (host)	
			RAB11A (host)	
			SNAP23 (host)	
			CDC42 (host)	
		hcmv-miR-UL112-3p	TLR-2 (host)	[91]
		hcmv-miR-UL112-3p	IKKα (host) IKKβ (host)	[92]
		hcmv-miR-US5-1	IKKα (host) IKKβ (host)	[92]
		hcmv-miR-US33as-5p	IFNAR1 (host)	[93]
		ebv-miR-BART16	CBP (host)	[94]
		ebv-miR-BART6-3p	RIG-I (host)	[95]
		ebv-miR-BART15-3p	NLRP3 (host)	[96]
		kshv-miR-K12-4-3p	ICAM1 (host)	[97]
		kshv-miR-K12-10a-3p	ICAM1 (host)	[97]
		kshv-miR-K12-9-3p	IRAK1 (host)	[98]
		kshv-miR-K12-5-3p	MYD88 (host)	[98]
		kshv-miRK12-11-3p	IKKε (host)	[99]
Promotion of reactivation	Viral	hcmv-miR-US22-5p	EGR1 (host)	[29]
		hcmv-miR-US5-2-5p	GAB1 (host)	[104]
	Cellular	hsa-miR-200b-3p	ZEB1 (viral) ZEB2 (viral)	[105]
		hsa-miR-429	ZEB1(viral) ZEB2 (viral)	[105]

N.D. = not determined.

## 11. Conclusions and Future Perspectives

From the various ways in which miRNAs regulate herpesvirus latency, some patterns can be observed. Most herpesviruses express miRNAs to restrict the lytic cycle during latency. Some directly target important viral lytic genes, and some indirectly repress lytic replication by regulating host pathways. Herpesviruses also generally utilize cellular miRNAs to restrict the lytic program. Interestingly, most of these mechanisms directly or indirectly lead to repression of viral IE gene expression (Figure 2), highlighting the necessity to block the initial activation of the lytic cycle for establishment of latency. Other mechanisms differ among different subfamilies. For instance, while regulating cell proliferation is an important function of many gammaherpesvirus miRNAs and some betaherpesvirus miRNAs, such mechanisms are irrelevant for alphaherpesviruses, which undergo latency in non-dividing cells. In addition, although beta and gamma herpesvirus miRNAs often block immune responses to promote persistence of the host cell during latency, there is little evidence that this is the case for alphaherpesvirus miRNAs. While fewer studies with alphaherpesvirus miRNAs could be one reason, it is also possible is that latently infected neurons may not be subjected to as strong immune surveillance as the cells harboring beta and gamma herpesviruses and that certain levels of immune surveillance in neurons might even favor latency. Another interesting pattern is that the main theme of miRNA regulation of herpesvirus latency seems to promote establishment and maintenance of latency rather than exit from it (reactivation). This is understandable, as the reactivation stimulus that allows lytic gene expression to surpass a certain threshold requires a fast process, but miRNAs are usually slow regulators.

Much has yet to be learned about the role of miRNAs in herpesvirus latency. Most work has focused on HSV, HCMV, EBV, and KSHV so far, leaving the other human herpesviruses largely unexplored. In addition, more work has focused on viral miRNAs than host miRNAs. We expect that much more about cellular miRNAs has yet to be discovered because as long as a virus needs to persist in a host, the cellular environment heavily influenced by thousands of cellular miRNAs needs to be adapted to or dealt with. There have been studies about changes in cellular miRNA expression profiles caused by herpesvirus infections [107]. However, few of these studies used experimental models that are relevant to latency, and the functions of differentially expressed miRNAs are often poorly defined. Another method for systematic identification of cellular miRNAs regulating herpesvirus latency is functional screening using a library of miRNA mimics or inhibitors, which, to our knowledge, has not been performed with herpesviruses but is likely to be useful in future.

Our understanding of the role of miRNAs in herpesvirus latency relies considerably on technical advances. First, experimental models of latency are crucial for elucidating the functions of miRNAs. HSV is the only human herpesvirus for which small animal models of latency are readily available. Mouse or rat primary neuronal culture models have also proven valuable in understanding molecular mechanisms of HSV latency and reactivation [108]. A major concern, though, is whether these models accurately recapitulate events occurring in humans. To solve this problem, promising models based on differentiated human neuronal cultures have recently been developed [109,110]. Other herpesviruses cannot easily infect small animals due to their strict host tropism, so in vivo studies for these viruses may require humanized mice. Alternatively, insight can be gained from studies of related animal viruses, such as MCMV and MHV-68, which are related to HCMV and gamma herpesviruses, respectively. Meanwhile, in vitro latency models of HCMV, EBV, and KSHV are available and very helpful to studies in this field.

Another set of techniques needed are those for identifying miRNA targets. Bioinformatics tools for target prediction have been greatly refined as we know more about the determinants of the efficacies of gene regulation by miRNAs [111]. Meanwhile, the last decade saw extensive application of genome-wide high-throughput experiments in herpesvirus research, including HITS-CLIP and PAR-CLIP [112,113,114,115,116]. With the development of the CLIP technology, optimized versions such as eCLIP [117] are likely to be powerful tools for profiling miRNA targetome, especially when used in combination with transcriptomic and proteomic approaches. Furthermore, cross-linking and sequencing of hybrids (CLASH) with the addition of a ligation step to the CLIP protocol has been applied to EBV-infected cells and may be used for other herpesviruses [118].

A fundamental remaining question is whether any of these miRNAs can be used to develop therapy against latent infection. We can imagine that if a certain miRNA is important for latency of a certain herpesvirus, we can potentially either introduce more of the miRNA to suppress reactivation or introduce its inhibitor to induce reactivation followed by antiviral drug treatment. However, development of such therapeutics is faced with the technical difficulty of delivering RNA sequences into latently infected cells, especially into neurons. Recent advances in small RNA delivery techniques may help solve this problem [119]. Another obstacle is the redundancy of miRNA functions, as reflected by the observation that the same pathways are often targeted by multiple miRNAs, making none of the individual miRNAs essential. Therefore, future studies need to elucidate the networks of miRNA-target interactions involved in herpesvirus latency so that we can perhaps use combinations of miRNAs or their inhibitors to interfere with the network. Inasmuch as miRNAs play an important role in herpesvirus latency, development of miRNA-based therapeutics remains promising and is worth exploring in future.

## Data Availability

Not applicable.
